# Functional Magnetic Mesoporous Silica Microparticles Capped with an Azo-Derivative: A Promising Colon Drug Delivery Device

**DOI:** 10.3390/molecules23020375

**Published:** 2018-02-10

**Authors:** Adrián H. Teruel, Carmen Coll, Ana M. Costero, Daniel Ferri, Margarita Parra, Pablo Gaviña, Marta González-Álvarez, Virginia Merino, M. Dolores Marcos, Ramón Martínez-Máñez, Félix Sancenón

**Affiliations:** 1 Interuniversity Research Institute for Molecular Recognition and Technological Development (IDM), Polytechnic University of Valencia, University of Valencia, 46100 Valencia, Spain; adherte@upv.es (A.H.T.); macolme1@upvnet.upv.es (C.C.); ana.costero@uv.es (A.M.C.); daniel.ferri@uv.es (D.F.); margarita.parra@uv.es (M.P.); pablo.gavina@uv.es (P.G.); Virginia.merino@uv.es (V.M.); mmarcos@qim.upv.es (M.D.M.); fsanceno@upvnet.upv.es (F.S.); 2 CIBER of Bioengineering, Biomaterials and Nanomedicine (CIBER-BBN), 28029 Madrid, Spain; 3 Department of Organic Chemistry, University of Valencia, 46100 Valencia, Spain; 4 Engineering Department. Pharmacy and Pharmaceutical Technology Section, Miguel Hernandez University, 03550 Alicante, Spain; marta.gonzalez@umh.es; 5 Pharmacy and Pharmaceutical Technology and Parasitology, University of Valencia, 46100 Valencia, Spain; 6 Joint Research Unit in Nanomedicine and Sensors. Polytechnic University of Valencia, Health Research Institute Hospital La Fe, 46100 Valencia, Spain.; 7 Joint Unit CIPF-UPV of Mechanisms of Diseases and Nanomedicine, Valencia, Polytechnic University of Valencia, Prince Felipe Research Center, 46100 Valencia, Spain

**Keywords:** magnetic mesoporous silica, azo derivatives, pH triggered, azo reductor, colon release

## Abstract

Magnetic micro-sized mesoporous silica particles were used for the preparation of a gated material able to release an entrapped cargo in the presence of an azo-reducing agent and, to some extent, at acidic pH. The magnetic mesoporous microparticles were loaded with safranin O and the external surface was functionalized with an azo derivative **1** (bearing a carbamate linkage) yielding solid **S1**. Aqueous suspensions of **S1** at pH 7.4 showed negligible safranin O release due to the presence of the bulky azo derivative attached onto the external surface of the inorganic scaffold. However, in the presence of sodium dithionite (azoreductive agent), a remarkable safranin O delivery was observed. At acidic pH, a certain safranin O release from **S1** was also found. The pH-triggered safranin O delivery was ascribed to the acid-induced hydrolysis of the carbamate moiety that linked the bulky azo derivatives onto the mesoporous inorganic magnetic support. The controlled release behavior of **S1** was also tested using a model that simulated the gastro intestinal tract.

## 1. Introduction

In recent years, the blending of coordination, molecular, supramolecular and biomolecular chemistry concepts with porous materials has led to the development of smart functional gated materials with multiple applications in different scientific and technological fields [1]. These hybrid materials are mainly composed by two subunits: (i) an inorganic porous support for cargo loading; and (ii) certain molecules acting as “molecular gates” for controlled cargo release upon the application of external stimuli [2].

One of the most commonly used inorganic supports in gated materials is mesoporous silica due to its outstanding features such as biocompatibility, inertness and chemical stability, high loading capacity and ease of functionalization using the well-known alkoxysilane chemistries [3]. Besides, mesoporous silica can be prepared as micro- or nanoparticles, the diameter of the pores can also be adjusted from the typical 2–3 nm range to ca. 6–10 nm (to include inside the porous network biomolecules such as small peptides, DNA fragments or enzymes) and can be prepared including other nanoparticles (for example, magnetite) in their structure endowing the final support with novel features [4,5,6].

These “gated materials” have been extensively used in controlled release processes [7,8,9,10] as well as in sensing/recognition protocols (when a dye or fluorophore is released in the presence of a target molecule) [11] and, very recently, in abiotic communication networks involving several gated nanodevices [12,13]. Dealing with controlled drug release, the preparation of carriers able to deliver certain drugs at-will and on the site of action, minimizing secondary effects, is a benchmark in the treatment of different diseases [14].

From another point of view, colon drug delivery systems (CDDS) have been developed for their use in two main applications: (i) to deliver macromolecular drugs, such as protein or peptides that found in the colon a better environment to be absorbed without losing activity because of the relatively low proteolytic enzyme activity and good absorption (e.g., insulin, calcitonin, and vasopressin); and (ii) to increase the efficacy in prevention and treatment of colon related diseases (e.g., irritable bowel syndrome, inflammatory bowel diseases (IBD), and colorectal cancer) [15,16]. In fact, the development of CDDS is an excellent strategy to deliver certain drugs in colon, decreasing adverse effects and enhance drug efficacy. With this aim, smart formulations for targeting colon such as pH-responsive biodegradable polymers, time-dependent formulations, pressure responsive systems, osmotic controlled materials, bio-adhesive systems, enzyme triggered prodrugs or drugs coated with enzyme-sensitive polymers have been recently developed [17,18,19].

Despite these examples, the use of hybrid organic–inorganic gated materials based on mesoporous silica for the development of CDDS is still an almost unexplored field. For the preparation of these CDDS silica-based nanodevices, enzymes produced by colon microbiota (azoreductase, β-galactosidase, β-xylosidase, nitroreductase, glycosidase deaminase, etc.) are ideal candidates to be used as external triggers [20,21].

Bearing in mind our interest in the development of silica-based gated materials for controlled release applications [22,23,24,25,26,27,28,29], we report herein the preparation of capped a micro-sized silica mesoporous solid containing magnetic nanoparticles (**S1**) as a suitable carrier to release an entrapped cargo in colon. Although not specifically tested in this present work, the magnetic character of the prepared nanodevice may be useful to enhance the retention time of the particles in the part of interest of the intestinal tract (for instance in colon). Moreover, particles **S1** are loaded with a dye and capped with an azoderivative attached to the external surface of the inorganic matrix by means of a carbamate group. We demonstrated that the presence of the bulky azoderivative onto the external surface of **S1** inhibited cargo release, whereas in the presence of a reducing agent delivery was observed. The solid was also able to deliver the cargo to some extent at acidic pH. A study on payload release from **S1** in a simulated digestive process in mouth, stomach, small intestine and colon is also reported.

## 2. Results and Discussion

### 2.1. Synthesis of Gated Magnetic Micro-Sized Mesoporous Silica Particles

**Scheme 1:**
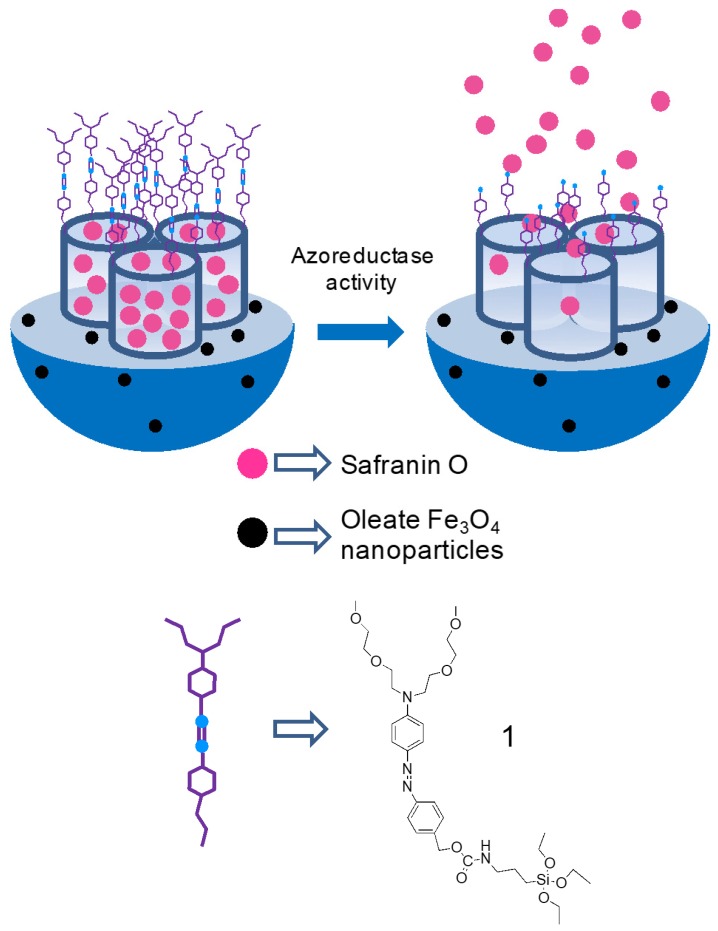
Magnetic micrometric silica mesoporous supports loaded with safranin O and capped with azo derivative (**1**). Safranin O is released in the presence of an azoreductor.

Magnetic nanoparticles (MNPs) coated with oleic acid were prepared by a co-precipitation procedure that used a mixture of FeCl_3_ and FeCl_2_ and ammonium hydroxide [30]. Moreover, mesoporous silica was prepared following the “atrane route” with small modifications [31,32]. In this synthetic protocol, *n*-cetyltrimethylammonium bromide was used as structural directing agent and tetraethylortosilicate as silica source. MNPs were suspended in water and incorporated into the synthesis crude before the addition of NaOH. The resulting brownish powder was washed and the surfactant was subsequently removed by calcination. This procedure yielded the final magnetic micro-sized inorganic solid (**S0**). Once the magnetic mesoporous support was synthesized, the pores of the siliceous phase were loaded with safranin O (as a model drug in our study) and the external surface functionalized with the bulky azo-containing derivative **1**. This synthetic protocol yielded micro-sized magnetic solids **S1** (see Scheme 1). It was expected that the bulky azo-containing derivative **1** would inhibit dye leaching from the pores, whereas safranin O delivery should be observed in the presence of sodium dithionite (able to hydrolyze azo linkages) or partially at acidic pH (due to the hydrolysis of the carbamate moiety in **1**).

**Scheme 2:**
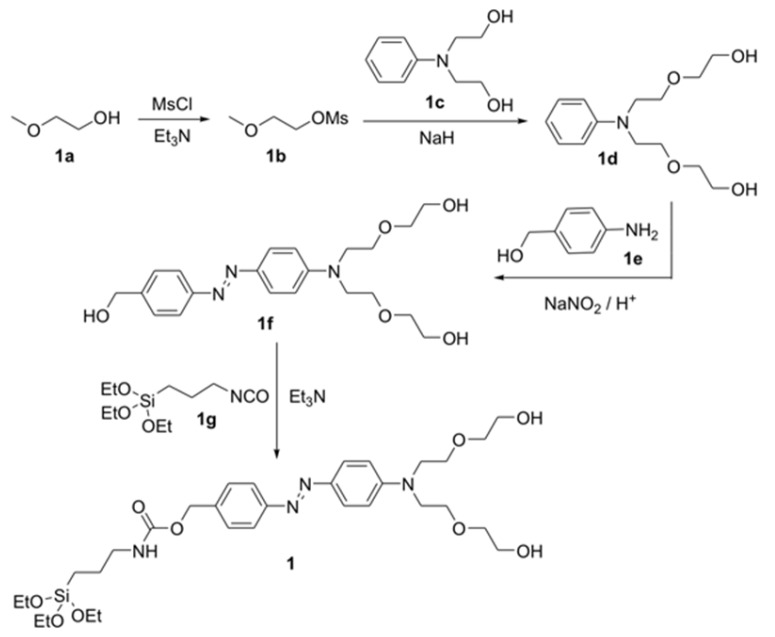
Synthetic route used for the preparation of azoderivative **1**. 2-methoxyethanol (**1a**); 2-methoxyethyl methanesulfonate (**1b**); *N*-phenyldiethanolamine (**1c**); oligoethylenglycol derivative (**1d**); 4-aminobenzyl alcohol (**1e**); azo derivative (**1f**); (3-isocyanatopropyl) triethoxysilane (**1g**).

The synthesis of azo-containing alkoxysilane derivative **1** (see Scheme 2) starts with the mesylation of 2-methoxyethanol (**1a**) in dichloromethane that yielded **1b [33]**. Then, in a second step, **1b** was reacted with the disodium salt of *N*-phenyldiethanolamine (**1c**) obtaining the oligoethylenglycol derivative **1d**. Afterwards, **1d** was reacted with the diazonium salt of 4-aminobenzyl alcohol (**1e**) yielding azo derivative **1f**. Finally, azo-containing trialkoxysilane derivative **1** was obtained upon reaction between **1f** and (3-isocyanatopropyl) triethoxysilane (**1g**). The final azo-containing trilakoxysilane derivative (**1**) was fully characterized using ^1^H- and ^13^C-NMR and HRMS.

### 2.2. Characterization of the Prepared Materials 

**Figure 1:**
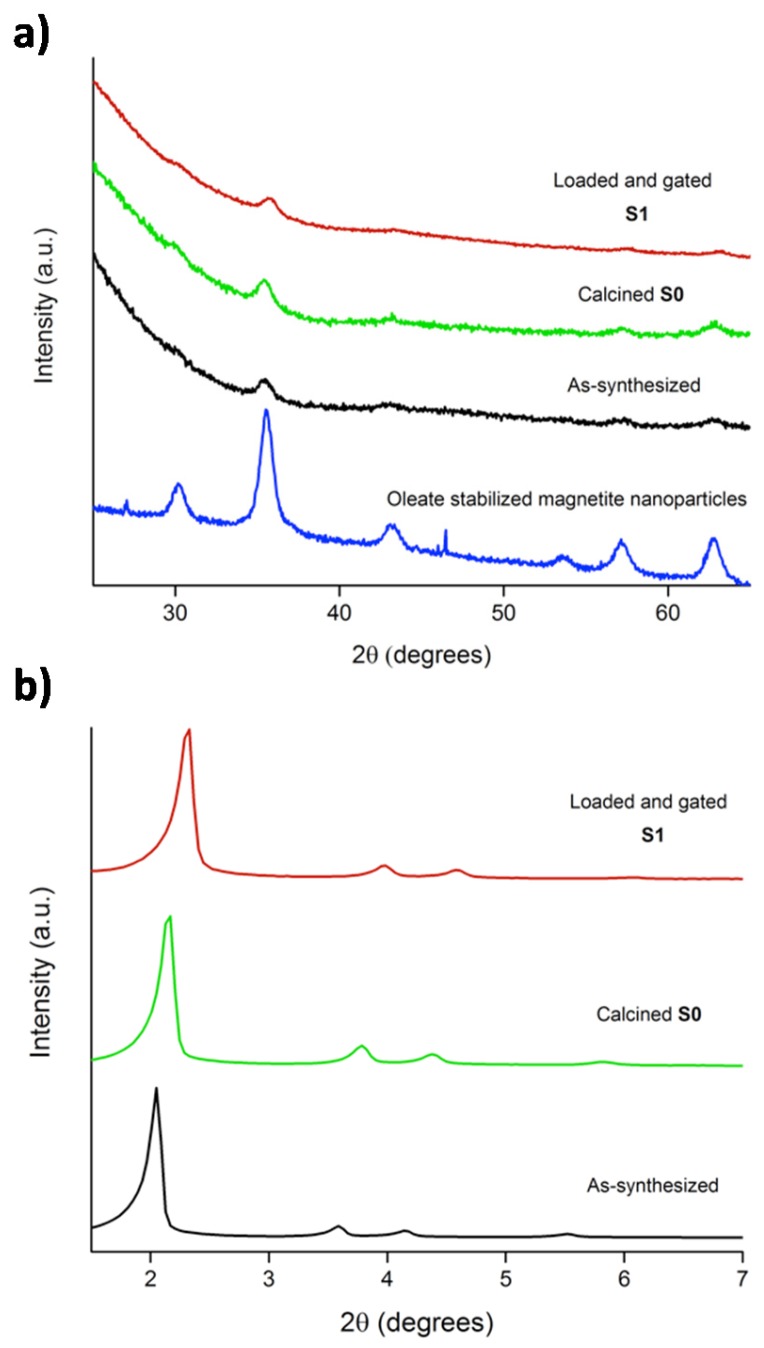
Powder X-rays patterns at: (**a**) high angles; and (**b**) low angles of oleate stabilized magnetite (Fe_3_O_4_) nanoparticles and magnetic micrometric mesoporous silica particles as-synthesized, **S0** and **S1**.

**S0** and **S1** were characterized using powder X-ray diffraction (PXRD), transmission electron microscopy (TEM), scanning transmission electron microscopy (STEM), N_2_ adsorption–desorption isotherms and thermogravimetric analysis. Figure 1 shows the PXRD patterns (at high and low angles) of oleate-stabilized MNPs, the magnetic mesoporous microparticles as-made, **S0** and **S1**. PXRD of oleate-coated MNPs (see also Figure 1) showed the five typical high-angle reflections of magnetite phase indexed as (220), (311), (400), (511) and (440) Bragg peaks. The same peaks were observed for the as-made microparticles, **S0** and **S1** materials but with a marked decrease in intensity (Figure 1). This intensity loss was ascribed to the inclusion of MNPs into the inorganic mesoporous matrix. Low angle PXRD pattern of as-made microparticles (Figure 1) displayed the typical reflections of a hexagonal-ordered mesoporous system that can be indexed as (100), (110), (200) and (210) Bragg peaks. A shift of the (100) peak in calcined **S0** solid was clearly observed. This displacement is consistent with an approximate cell contraction of 3.64 Å and is attributed to the condensation of silanol groups during the calcination step. The presence of the characteristic (100) reflection in the diffraction spectrum of **S1** clearly indicated that the mesoporous structure was preserved throughout the filling process with safranin O and the anchoring of the azo derivative **1**.

**Figure 2:**
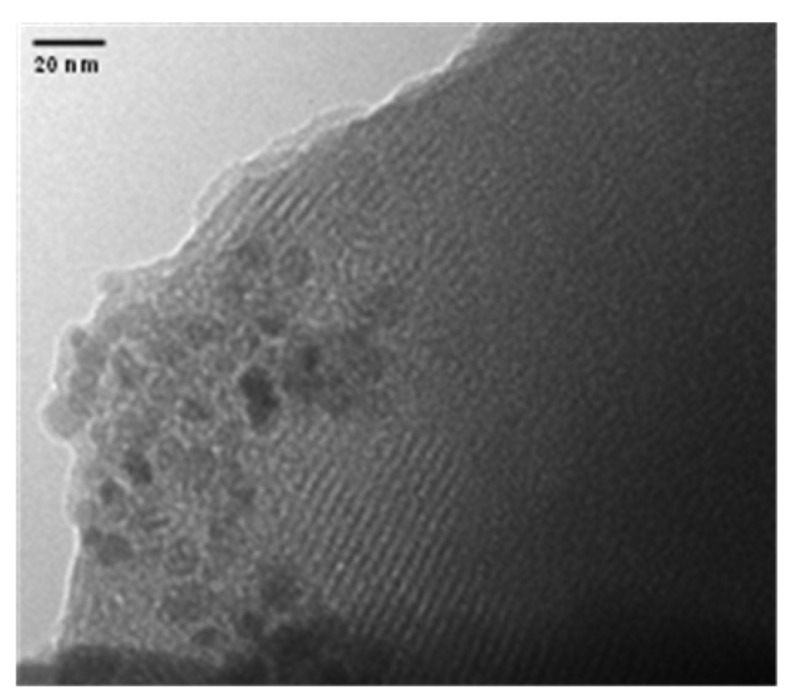
TEM image of micrometric mesoporous silica particles containing magnetite nanoparticles (**S0**).

**Figure 3:**
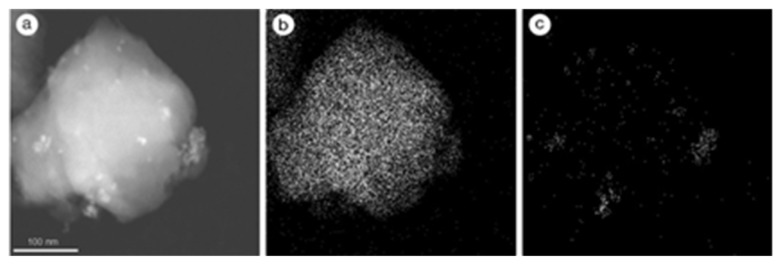
(**a**) STEM images of micrometric mesoporous silica particles containing magnetite nanoparticles (**S0**); (**b**) silica mapping; and (**c**) iron mapping.

**Figure 4:**
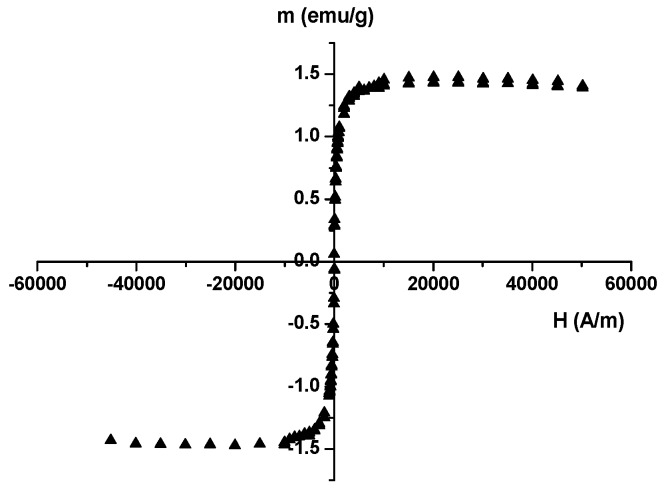
Field-dependent magnetization curves at room temperature of **S0** microparticles.

Figure 2 shows transmission electron microscopy (TEM) images of solid **S0**. As could be seen, the typical porosity associated with the mesoporous inorganic support was present. Besides, MNPs were observed as small dark dots. Scanning transmission electron microscopy (STEM) images of solid **S0** (iron mapping) indicated that MNPs are randomly distributed through the mesoporous silica microparticles (Figure 3). Moreover, to assess the magnetic behavior of **S0**, field dependent magnetization curves measured at 298 K were obtained. The obtained curves (see Figure 4) displayed no hysteresis loops and showed coercivity values near zero, in agreement with a typical superparamagnetic behavior at room temperature. The saturation magnetization value for **S0** was 1.5 emu/g. This magnetization value is not far from those reported in the literature for other magnetic mesoporous silica particles, which are typically in the 1.7–10 emu/g range [30].

**Figure 5:**
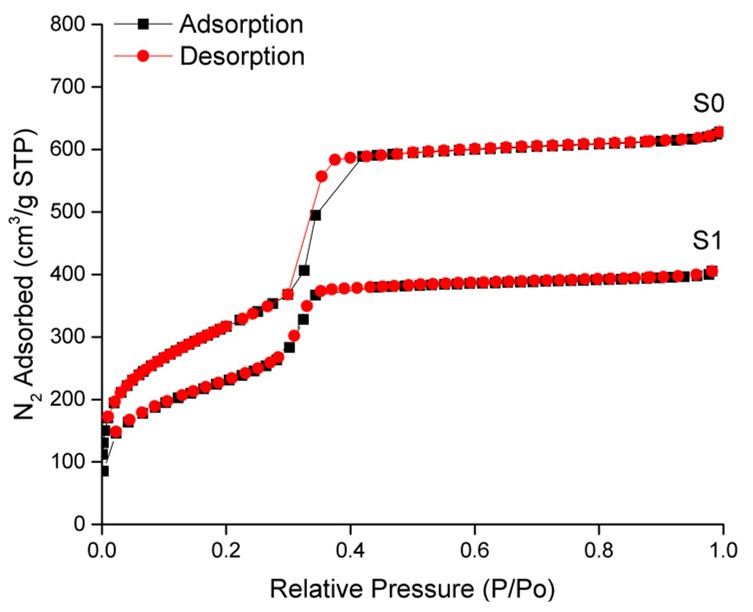
N_2_ adsorption–desorption isotherms for **S0** and **S1** materials.

N_2_ adsorption–desorption studies of **S0** and **S1** were also carried out (Figure 5). As could be seen, solid **S0** showed a typical curve for mesoporous silica materials, with an adsorption step at intermediate P/P_0_ values (0.2–0.4). This isotherm is classified as type IV in which the absorption step deals with nitrogen condensation inside the mesopores. The pore diameter of **S0** was calculated by the Barret-Joyner-Halenda (BJH) method [34]. The narrow BJH pore distribution observed and the absence of a hysteresis loop in the 0.2–0.4 P/P_0_ interval suggested the existence of uniform cylindrical mesopores. Values of pore diameter of 2.83 nm and pore volume of 0.94 cm^3^ g^−1^, calculated on the adsorption branch of the isotherm, were found. Pore diameter estimated from TEM images agree with this value. The application of the Brunauer-Emmett-Teller (BET) model [35] gave a value of 1057 m^2^ g^−1^ for the total specific surface. On the other hand, the N_2_ adsorption–desorption isotherms of solid **S1** are typical of mesoporous systems with partially filled mesopores, and a decrease in both the adsorbed N_2_ volume and the specific surface area was clearly observed (Table 1). This reduction in the BET surface, compared with that of **S0**, was ascribed to the loading of pores with safranin O and the functionalization of the surface with the bulky azo derivative **1** in **S1**.

Total organic matter on solid **S1** was determined by thermogravimetric analysis (see Supplementary Materials) and ^1^H-NMR (Table 2). The amount of anchored azo derivative onto **S1** was determined by ^1^H-NMR upon dissolving the corresponding sample in HF/D_2_O in the presence of tetraethyl ammonium bromide as internal standard (Table 2) [36]. Besides, energy dispersive X-ray spectroscopy (EDX, 20 kV) was used to determine Si and Fe content in **S1** microparticles. EDX measurements showed a value of 5.9 for the Si/Fe ratio in the final **S1** solid.

### 2.3. Cargo Release from ***S1***

**Figure 6:**
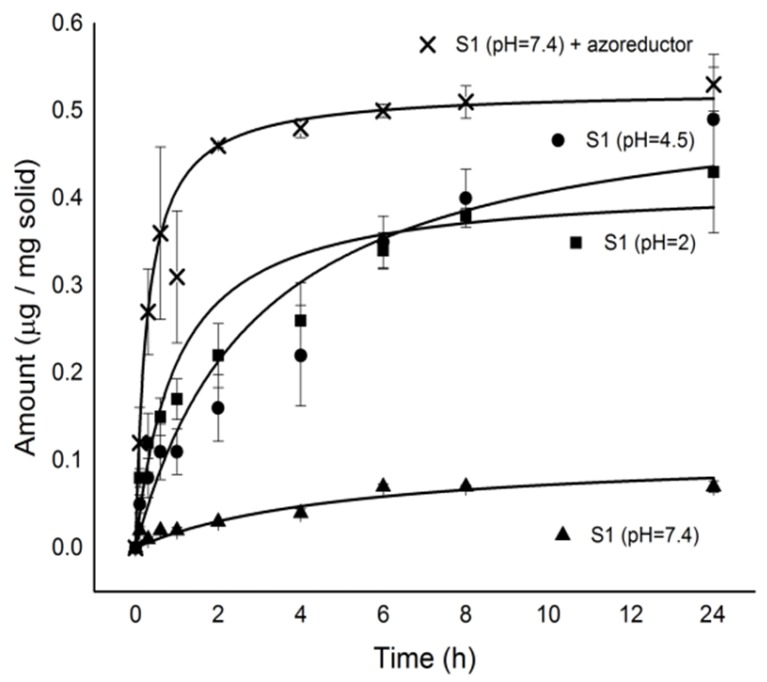
Release kinetics of safranin O from **S1** in water at pH ≈ 2 (square), pH ≈ 4.5 (circle), pH ≈ 7.4 (triangle) and pH ≈ 7.4 in the presence of azoreductor agent sodium dithionite (SD, 57.4 mM) (cross).

Payload release from solid **S1** at acidic (2.0 and 4.5) and neutral (7.4) pH in the presence or absence of the azoreductor agent sodium dithionite was studied [37,38]. These pH values were selected taking into account the possible use of **S1** as nanodevice for controlled release in colon: pH 2.0 is typical of gastric juices, pH 4.5 is found in the transition from stomach to intestines (this pH can also be found in the colon of IBD patients), and a neutral pH ranging 6.5–7.4 is typically found in the intestine. In a typical experiment, 2 mg of **S1** were suspended in water at selected pH values, the final suspensions stirred at 37.5 °C and aliquots were taken at scheduled times. The aliquots were centrifuged to remove the solid and the release of safranin O to the solution monitored by measuring fluorescence at 571 nm upon excitation at 520 nm. Cargo release profile for solid **S1** is shown in Figure 6. Aqueous suspensions of **S1** showed negligible safranin O release at neutral pH. However, as a clear contrast, a marked delivery of entrapped safranin O was seen in the presence of sodium dithionite (more than 80% of maximum dye delivered after 4 h). The observed delivery is attributed to the presence of sodium dithionite, which can reduce azo groups in the capping molecule **1** resulting in pore opening and safranin O release [37,38]. At acidic pH (2.0 and 4.5), a sustained safranin O release was also observed. Delivery of entrapped safranin O was ascribed to a hydrolysis of carbamate moieties, which linked the bulky azo derivative **1** onto the external surface of **S1**. Maximum release of safranin O for **S1** was ca. 0.51 μg/mg solid at pH 7.4 in the presence of sodium dithionite.

### 2.4. In Vitro Digestion Model Assay

**Figure 7:**
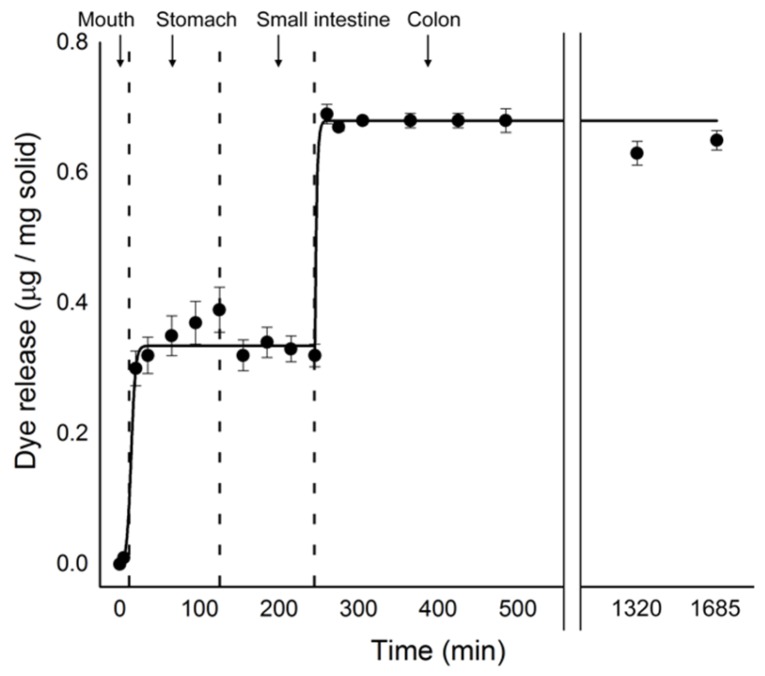
Release kinetics of Safranin O from **S1** in simulated gastro-intestinal tract (GIT) fluids.

Despite the partial payload delivery from **S1** at acidic pH shown above, it has to be noted that permanence of the particles in the stomach is usually less than 2 h and therefore only a partial cargo delivery from **S1** is expected to occur before targeting colonic tissues. In fact, to study in depth the possible application of azo-gated magnetic microparticles as suitable particles for cargo delivery in colon, payload delivery from **S1** was further tested in a model of digestion using simulated solutions. The model used was a modification of that introduced by Oomen and co-workers [39,40] that is a three-step procedure simulating digestive process in mouth, stomach and small intestine. Moreover, we introduced a fourth step to simulate the digestive process in colon. In a typical experiment, 2 mg of solid (**S1**) were suspended in simulated saliva (5 min at 37.5 °C) and then simulated gastric juice (stomach) was added and the mixture stirred 2 h. Afterwards, duodenal simulated juice, bile and bicarbonate solution (small intestine) were added and the suspension stirred for two more hours. Finally, sodium dithionite was added and it was left for 24 h to mimic the colon environment and the presence of azoreductases. Safranin O release in the entire process was evaluated via monitorization of the emission at 571 nm upon excitation at 520 nm. The obtained results are shown in Figure 7.

As shown in Figure 7, a nearly “zero release” was observed during the first 5 min when solids were in contact with simulated saliva. In contact with gastric juice, a safranin O delivery of ca. 0.3 μg/mg solid was found and ascribed to partial pore opening due to the acidic environment. In small intestine conditions, no further delivery of safranin O was observed. However, a marked cargo release was found in simulated colon conditions and related with the presence of sodium dithionite, which can reduce azo bonds of the capping ensemble in **S1**.

## 3. Materials and Methods 

### 3.1. General Techniques 

Transmission electron microscopy (TEM), scanning transmission electron microscope (STEM), powder X-ray diffraction (PXRD), thermogravimetric analysis (TGA), N_2_ adsorption–desorption, nuclear magnetic resonance (NMR), Fourier transform infrared spectroscopy (FT-IR) and fluorescence spectroscopy techniques were employed to characterize the synthesized materials. TEM and STEM images were acquired using a JEOL JEM-1010 and a JEOL JEM 2100F microscopes (Akishima, Japan), respectively. PXRD measurements were performed on a Bruker D8 Advance diffractometer (Billerica, MA, USA) using CuK_α_ radiation. TGA were carried out on a TGA/SDTA 851e Mettler Toledo balance (Greifensee, Switzerland), using an oxidant atmosphere (air, 80 mL/min) with a heating program consisting on a heating ramp of 10 °C per minute from 393 to 1273 K and an isothermal heating step at this temperature for 30 min. N_2_ adsorption–desorption isotherms were recorded with a Micromeritics ASAP 2010 automated sorption analyzer (Norcross, GA, USA). The samples were degassed at 120 °C under vacuum overnight. The specific surface areas were calculated from the adsorption data in the low pressure range using the Brunauer-Emmett-Teller (BET) model. Pore size was determined following the Barrett-Joyner-Halenda (BJH) method. ^1^H NMR spectra were recorded using a Bruker AV400 spectrometer (Billerica, MA, USA). Infrared spectra were recorded using a Bruker Tensor 27 equipment (Billerica, MA, USA). Fluorescence measurements were carried out in a PerkinElmer EnSpire Multimode Plate Reader (Waltham, MA, USA).

### 3.2. Chemicals 

The chemicals tetraethylorthosilicate (TEOS), *n*-cetyltrimethylammonium bromide (CTABr), sodium hydroxide, triethanolamine (TEAH_3_), (3-isocyanatopropyl) triethoxysilane, iron(III) chloride hexahydrate, iron(II) chloride tetrahydrate, oleic acid, safranin O dye, 2-methoxyethanol, triethylamine, methanesulfonyl chloride, hydrochloric acid, *N*-phenyldiethanolamine, anhydrous acetonitrile, sodium hydride, 4-aminobenzyl alcohol, sodium nitrite, sodium dithionite and all chemicals for the digestive fluids were provided by Sigma-Aldrich (Madrid, Spain). Magnesium sulfate, sodium carbonate, and all the analytical-grade solvents were purchased from Scharlab (Barcelona, Spain). Aluminum oxide was acquired from Merck (Madrid, Spain). All products were used as received.

### 3.3. Synthesis of Oleic Acid-coated Magnetic Nanoparticles (MNPs) 

MNPs were prepared using a modified co-precipitation method. In a typical procedure, FeCl_3_·6H_2_O (24.0 g, 0.089 mol) and FeCl_2_·4H_2_O (9.8 g, 0.049 mol) were dissolved in deionized water (100 mL) under nitrogen atmosphere with vigorous stirring at 80 °C. Then ammonium hydroxide (50 mL) was added quickly into the solution. The colour of the solution immediately turned black and then oleic acid (3.8 g, 0.013 mol) was added after 30 min. The reaction was kept at 80 °C for 1.5 h. The final oleic acid-coated MNPs were washed with deionized water until neutral pH. Afterwards, the MNPs were transferred into a chloroform solution (30 mg MNPs/mL CHCl_3_).

### 3.4. Synthesis of Mesoporous Silica Microparticles Containing MNPs (***S0***) 

A chloroform suspension of MNPs (6 mL, 30 mg MNPs/mL CHCl_3_) was mixed with an aqueous solution of CTABr (8 mL, 10 mg/mL) and the resulting suspension was treated with ultrasounds and heated. Finally, ultrasounds were applied again and the suspension was passed through nylon filters (0.45 μM) to ensure MNPs homogeneity. Magnetic micro-sized MCM-41 particles were synthesized following the so-called “atrane route”. For this purpose, a solution of TEAH_3_ (25.79 g, 0.173 mol) and NaOH (2 mL of a 6 M solution) was heated to 120 °C and then cooled down to 70 °C, at this moment TEOS (11 mL, 0.045 mol) was added and the crude reaction was heated up to 120 °C. The crude reaction was left cooling down again and CTABr (4.68 g, 0.013 mol) was added at 118 °C. Next, water containing MNPs (74 mL of distilled water + 6 mL of the MNPs CTABr-water suspension) was slowly added with vigorous stirring at 70 °C. Besides, NaOH (10 mL of a 0.1 M solution) was added to obtain a pH ≈ 10.9. After a few minutes, a brownish suspension was formed. This mixture was aged in an autoclave at 100 °C for 24 h. The resulting powder was collected by filtration and washed with water. Finally, the solid was dried at 70 °C. To prepare the final solid (**S0**), the as-synthesized microparticles were calcined at 550 °C using oxidant atmosphere for 5 h to remove the template.

### 3.5. Synthesis of ***1b***

2-Methoxyethanol (**1a**, 16 mL, 0.2 mol) was dissolved in dichloromethane (270 mL) in a 2 L round-bottomed flask. The solution was kept in an ice bath for 15 min, and then triethylamine (55.2 mL, 0.4 mol) was added to the solution. Afterwards, a solution of methanesulfonyl chloride in dichloromethane (0.32 mol in 48 mL) was added dropwise for 60 min to the initial reaction crude. After this addition, reaction was stirred at room temperature for another 90 min period. Then, the crude was poured onto a water/ice mixture containing concentrated hydrochloric acid (50 mL), and the organic layer was separated, washed three times with brine and dried with anhydrous MgSO_4_. Dichloromethane was eliminated in a rotary evaporator to give **1b** as yellow oil (25.16 g, 0.18 mol, 91% yield). Spectroscopic data were coincident with those reported in the literature.

### 3.6. Synthesis of ***1d***

*N*-phenyldiethanolamine (**1c**, 3.90 g, 0.021 mol) was dissolved in dry acetonitrile (70 mL) and then the flask was purged several times with argon to remove oxygen and water. Then, sodium hydride (1.2 g, 0.05 mol) was gradually added at room temperature, after which a white precipitate appeared. On the other hand, **1b** (6 g, 0.043 mol) was dissolved in anhydrous acetonitrile (30 mL) and then added dropwise to the generated dianionic salt of **1c**. After this addition, the crude reaction was heated at reflux for 24 h. Then, the reaction mixture was filtered off and the organic solvent was eliminated using a rotary evaporator, yielding a yellow oil. The product **1d** (1.25 g, 4 mmol, 20% yield) was purified using column chromatography with aluminum oxide and hexane/ethyl acetate (8:2 *v*/*v*) as the eluent. Spectroscopic data were consistent with those reported in the literature.

### 3.7. Synthesis of ***1f***

4-aminobenzyl alcohol (**1e**, 414 mg, 3.36 mmol) was dissolved in hydrochloric acid (150 mL, 4 mol L^−1^). This solution was slowly added to water (25 mL) containing sodium nitrite (232 mg, 3.36 mmol) at 5 °C. The generated diazonium salt of **1e** was immediately added to an aqueous solution containing **1d** (1 g, 3.36 mmol). The reaction was allowed to react for 30 min at 5 °C and for 30 min at room temperature. The final dark red crude was neutralized with a saturated sodium carbonate solution and the organic product was extracted with dichloromethane. Organic layers were dried with MgSO_4_ filtered off and the solvent was eliminated in a rotary evaporator. Product **1f** (250 mg, 0.58 mmol, 17% yield) was isolated as a dark red-orange solid through column chromatography with aluminum oxide and hexane/ethyl acetate (3:2 *v*/*v*) as the eluent. ^1^H-NMR (400 MHz, CDCl_3_): δ = 7.84 (d, 4H), 7.46 (d, 2H), 6.79 (d, 2H), 4.75 (s, 2H), 3.74–3.49 (m, 16H), 3.38 ppm (s, 6H); ^13^C: δ = 50.2, 57.4, 58.1, 67.3, 69.7, 70.9, 110.6, 121.2, 126.5, 127.4, 141.1, 154.9, 156.4 ppm.

### 3.8. Synthesis of ***1***

In a first step, **1f** (520 mg, 1.2 mmol) was dissolved in chloroform (20 mL). Then, excess of alkoxysilane derivative **1g** (468 µL, 1.8 mmol) and triethylamine (468 µL, 0.003 mmol) were added under argon atmosphere. The crude reaction was allowed to react for 72 h at 60 °C. Afterwards, the solvent was eliminated in a rotary evaporator and the reaction crude was washed 3 times with cold hexane to remove impurities. Compound **1** was isolated as orange oil (550 mg, 0.810 mmol, 67% yield). ^1^H-NMR (400 MHz, CDCl_3_): δ = 7.76 (d, 4H), 7.38 (d, 2H), 6.72 (d, 2H), 5.06 (s, 2H), 4.68 (s, 1H), 3.75 (q, 6H), 3.62–3.47 (m, 16H), 3.31 (s, 6H), 3.14 (t, 2H), 1.56 (m, 2H), 1.15 (t, 9H), 0.56 (t, 2H) ppm; ^13^C: δ = 160.9, 142.3, 139.4, 129.9, 128.5, 127.6, 122.4, 119.1, 114.2, 111.8, 100.2, 72.1, 70.8, 68.5, 65.3, 59.2, 51.4, 32.1, 29.9, 29.5, 22.8, 14.3, 1.2 ppm. HRMS-EI *m*/*z*: calcd for C_33_H_54_N_4_O_9_Si 678.3660; found: 677.2403 (M − H^+^).

### 3.9. Synthesis of ***S1***

Magnetic mesoporous silica microparticles (**S0**, 270 mg) were suspended in an acetonitrile solution (10 mL) of safranin O (75 mg, 0.8 mmol/g **S0**) under argon atmosphere. The obtained suspension was then stirred at room temperature overnight to achieve maximum loading of the pores. Afterward, **1** (550 mg, 3 mmol/g **S0**) was dissolved in anhydrous acetonitrile (8 mL) and was added to the microparticle/dye suspension. The mixture was stirred at room temperature for 6 h in an argon atmosphere. **S1** was isolated by centrifugation, washed several times with acetonitrile and was finally dried at 40 °C for 24 h.

### 3.10. Controlled Release Studies

Controlled release studies from **S1** were tested at different pH values (i.e., 2.0, 4.5 and 7.4) and in the presence of a reductive environment (sodium dithionite, which is known to reduce azo linkages). In a typical experiment, 2 mg of the corresponding solid were suspended in water at the selected pH (2 mL) and aliquots were extracted at given times (0, 5 min, 20 min, 40 min, 1 h, 2 h, 4 h, 6 h, 8 h, and 24 h), centrifuged to remove the solid and supernatants were loaded into a 96-well plate to measure safranin O fluorescence at 571 nm (excitation at 520 nm). Controlled release from **S1** microparticles were also tested in in vitro digestion conditions using simulated solutions for saliva, gastric juice, duodenal juice and bile [39,40]. In vitro digestion experiments were carried out with **S1** (2 mg) at 37 °C (temperature of the human body) and stirring at 12,000 rpm (to ensure the correct suspension of the microparticles). Digestions started by adding simulated saliva fluid (320 µL) and incubating for 5 min. Then, simulated gastric juice (630 µL) was added, and the mixture was stirred for 2 h. Later, simulated duodenal juice (630 µL), bile (320 µL) and a bicarbonate solution (1 M, 100 µL) were added simultaneously and the mixture was stirred for another 2 h. Finally, colon fluid was simulated by adding sodium dithionite (20 mg, 57.4 mM/2 mL). Aliquots were taken at given times (0, 5, 20, 35, 65, 95, 125, 155, 185, 215, 245, 260, 275, 305, 365, 425, 485, 1320 and 1685 min), centrifuged and supernatants were loaded into a 96 well plate to measure safranin O fluorescence. Calibration curves, in water, simulated small intestine fluid and simulated colon fluid, were performed to assess the amounts of safranin O released from solid **S1** in the different environments.

## 4. Conclusions

As summary, here we have reported the synthesis and characterization of magnetic mesoporous silica microparticles loaded with safranin O and capped with bulky azo derivative bearing pH-hydrolysable moieties (carbamate group). At neutral pH, the capped material showed a negligible safranin O release. However, at neutral pH, addition of a reducing agent such as sodium dithionite (mimicking azoreductase enzyme) induced a marked safranin O release. The observed delivery was ascribed to a reduction of the azo bonds that induced a fall in the steric crowding around pore outlets with subsequent dye delivery. Moreover, a partial payload release was also observed at acidic pH. Delivery studies in a digestion model indicated an important part of the payload was released in colon. These particles are expected to find applications as smart controlled drug delivery systems in the colon mucosa (e.g., for IBD patients) due to the presence of azoreductase enzymes produced by bacteria in the colon microbiota. Besides, its ability to release the cargo at acidic pH, opens the possibility to improve the performance of these carriers for drug delivery in ulcerative colitis and Crohn’s disease in which a marked decrease in the pH of the colon happens [41,42]. Moreover, these microparticles (**S1**) were equipped with magnetic nanoparticles to potentially apply magnetic fields to enhance the retention time of the solids in colon. Similar magnetic microparticles to those reported here and loaded with a drug (e.g., 5-ASA) are promising materials for the oral treatment of colon related diseases. Further in vivo studies in this line will be performed in due course.

## Figures and Tables

**Table 1:** BET specific surface values, pore volumes and pore sizes for **S0** and **S1**.

Solid	S_BET_ (m^2^ g^−1^)	pore volume ^1^ (cm^3^ g^−1^)	pore size ^1,2^ (nm)
**S0**	1057	0.94	2.83
**S1**	834	0.68	-

^1^ Total pore volume according to the BJH model. ^2^ Pore size estimated by using the BJH model applied on the adsorption branch of the isotherm, for P/P_0_ < 0.6, which can be associated to the surfactant generated mesopores.

**Table 2:** Content of total organic matter (in g) per gram of SiO_2_, content of azo derivative (in g) per gram of SiO_2_ and dye released (in µg) per mg of solid in **S1**.

Solid	Organic Content (g/g SiO_2_)	Azo Derivative (g/g SiO_2_)	Dye Release (µg/mg solid)
**S1**	0.0469	0.043	0.69

## Supplementary Materials

The Supplementary Materials are available online.
