# Supplementary material for: Functional Magnetic Mesoporous Silica Microparticles Capped with an Azo-Derivative: A Promising Colon Drug Delivery Device
Source: Molecules. 2018 Feb 10;23(2):375. doi: 10.3390/molecules23020375 (PMC6017295; doi:10.3390/molecules23020375)
Supplement: Supplementary file 1 [file molecules-23-00375-s001.pdf]

Supporting Information

Functional Magnetic Mesoporous Silica Microparticles Capped with an Azo-Derivative: a Promising Colon Drug Delivery Device

Adrián H. Teruel [1,2], Carmen Coll [1,2], Ana M. Costero [1,2,3], Daniel Ferri [1,3], Margarita Parra [1,2,3], Pablo Gaviña [1,2,3], Marta González-Álvarez [4], Virginia Merino [1,5], M. Dolores Marcos [1,2,6,7], Ramón Martínez-Máñez [1,2,6,7,*] and Félix Sancenón [1,2,6,7]

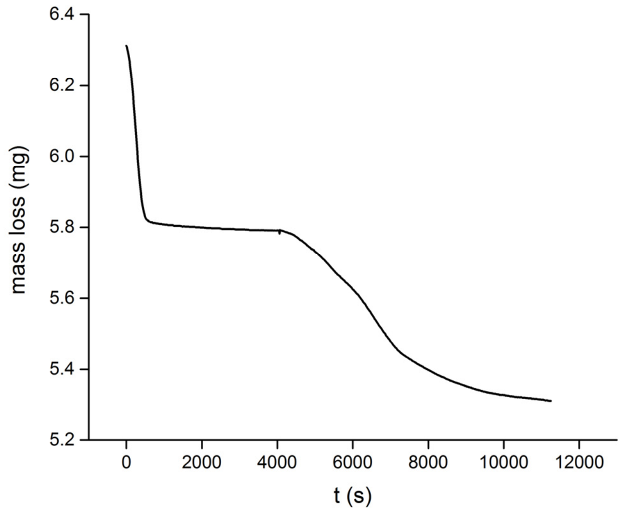

Figure SI-1. TGA curves of **S1** magnetic microparticles.

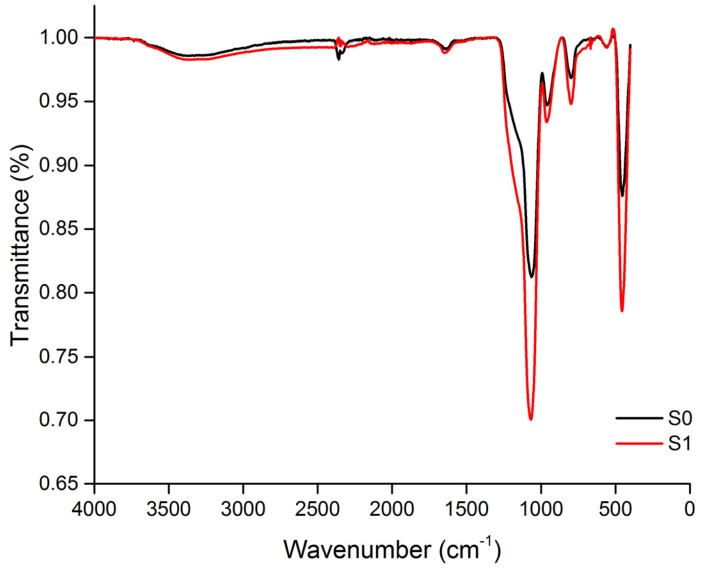

Figure SI-2. FTIR spectra of magnetic micro-sized inorganic solid (**S0**) and of the final loaded and capped microparticles (**S1**).
